# In vitro antimicrobial activities of animal-used quinoxaline 1,4-di-*N*-oxides against mycobacteria, mycoplasma and fungi

**DOI:** 10.1186/s12917-016-0812-7

**Published:** 2016-09-06

**Authors:** Yan Zhao, Guyue Cheng, Haihong Hao, Yuanhu Pan, Zhenli Liu, Menghong Dai, Zonghui Yuan

**Affiliations:** 1 National Reference Laboratory of Veterinary Drug Residues (HZAU) and MAO Key Laboratory for Detection of Veterinary Drug Residues, Huazhong Agricultural University, Wuhan, Hubei 430070 China; 2 MOA Laboratory for Risk Assessment of Quality and Safety of Livestock and Poultry Products, Huazhong Agricultural University, Wuhan, Hubei 430070 China; 3 Hubei Collaborative Innovation Center for Animal Nutrition and Feed Safety, Huazhong Agricultural University, Wuhan, Hubei 430070 China

**Keywords:** Quinoxaline 1,4-di-*N*-oxides, Antituberculosis, Antimycoplasma, Antifungi, Combined antimicrobial susceptibility test

## Abstract

**Background:**

The quinoxaline 1,4-di-*N*-oxides (QdNOs) were known as potent antibacterial agents. For the purpose of evaluating the bioactivity of existing animal-used QdNOs drugs against representative pathogenic microorganism, the representative drugs of quinoxalines including cyadox, mequindox, quinocetone and their metabolites were submitted to the in vitro evaluation for antituberculosis, antimycoplasma, antifungal and antiviral activities.

**Results:**

In antituberculosis assays, the prototype compounds were active (MIC = 4 ~ 8 μg/mL) against *Mycobacterium tuberculosis* H37Rv and *M. bovis.* Combined antimicrobial susceptibility test indicated that cyadox, mequindox and quinocetone combined with rifampicin had additive effect against *M. tuberculosis* complex with Fractional Inhibitory Concentration Index (FIC) of 0.75. Results of antifungal assays showed that quinocetone was active against *Microsporum canis* with MIC of 8 μg/mL. Antimycoplasma screening showed a generally good activity of quinocetone against *Mycoplasma gallisepticum* and *Mycoplasma hyopneumoniae*, with MIC between 8 and 16 μg/mL. As shown from the combined antimicrobial susceptibility test, cyadox, mequindox and quinocetone combined with tetracycline had additive effect against *Mycoplasma gallisepticum* with FIC of 0.75. These compounds were also submitted to antiviral assay against infectious bursal disease virus, porcine reproductive and respiratory syndrome virus, porcine parvovirus and classical swine fever virus. The results obtained showed that these QdNOs and their metabolites have no inhibitory activity against these viruses in vitro.

**Conclusions:**

QdNOs exhibit antimicrobial activities against mycobacteria, mycoplasma and fungi. This study gives new insight in further application of QdNOs and offers a way to promote the healthcare of animal husbandry.

**Electronic supplementary material:**

The online version of this article (doi:10.1186/s12917-016-0812-7) contains supplementary material, which is available to authorized users.

## Background

The microorganism infection is one of the most serious threats to human health and animal production all the time. With the help of antimicrobial agents, we have a powerful weapon against pathogens. However, the misuse of antimicrobials has led to the development of drug-resistant and multidrug-resistant (MDR) microorganisms [1]. Resistant bacteria are increasing and the interval between the appearances of new multi-drug resistant species is happening in short periods of time [2]. As MDR bacteria are increasing worldwide, development of new antimicrobials with enhanced activity is urgently needed [3]. In addition, it is a cost-effective approach to evaluate the bioactivity of existing drugs that can reverse the resistance and over turn the actual bacterial profile.

The quinoxaline 1,4-di-*N*-oxides (QdNOs) have been known as potent antibacterial agents since 1940s [4]. Animal-used QdNOs are a class of synthetic antibacterial agents, and the representative drugs, carbadox, olaquindox, mequindox (MEQ) and quinocetone (QCT) have been widely used in animal production as antibacterial growth promoters. Previous studies demonstrated that these drugs were active to many pathogenic microorganisms, including *Escherichia coli, Salmonella spp.*, *Staphylococcus aureus*, *Pasteurella multocida*, *Brachyspira hyodysenteriae*, etc. [5].Cyadox (CYA) is a new member of QdNOs, which may substitute olaquindox and carbadox because of its low toxicity and broad antibacterial spectrum [6–9]. Over the last two decades, many papers have been published, in which both synthesis and biological activity assessment of a large number of QdNOs derivatives have been described [10, 11]. Recent studies have demonstrated that QdNOs are endowed with antituberculosis [12, 13], antiviral [14], antichagasic [15], anticandida [16] activities and property of hypoxic selectivity [17], depending on specific chemical features. The previous encouraging results prompted us to further analyze the biological activity of the animal-used QdNOs.

For the purpose of obtaining new and more potent drugs which can improve the current chemotherapy against representative pathogenic microorganism, CYA, QCT, MEQ and their metabolites were evaluated for in vitro antimicrobial activity. The antimicrobial minimum inhibitory concentration (MIC) of QdNOs and their metabolites against fungi, mycoplasma and *Mycobacterium tuberculosis* complex were examined. Also, the inhibitory activity of QdNOs against infectious bursal disease virus (IBDV), porcine reproductive and respiratory syndrome virus (PRRSV) and porcine parvovirus (PPV) were evaluated by cytopathic effect (CPE) method and methyl thiazolyl tetrazolium (MTT) method. Since the replication of classical swine fever virus (CSFV) does not result in cytopathic effect in vitro, a SYBR Green I real-time RT-PCR was developed to determine the copies of the virus suspension. By comparing the growth curve, whether these QdNOs have anti-CSFV activity can be judged. Meanwhile, the combined antimicrobial susceptibility test were carried out in order to screen the drug combinations against *M. tuberculosis* complex and mycoplasma, providing the scientific basis for the further application of these drugs.

## Methods

### Bacteria, viruses and cells

The fungi including *Aspergillus fumigatus* 3.5301 and 3.5352*, Candida albicans* 2.4122 and 2.3990 (ATCC7349), *C. tropicalis* 2.1975 (ATCC7349) and 2.2735*, C. parapsilosis* 2.1846 (ATCC22019)*, Cryptococcus neoformans* 2.3201, *Trichophyton rubrum* ATCC4438 and CMCC(F)T1I, *Epidermophyton floccosum* CBS566094 and CMCC(F)E3D and *Microsporum canis* CMCC(F)M3D and CBS113480, and the mycoplasma including *Mycoplasma gallisepticum* BG44T (CVCC350) and PG31 (CVCC352) and *M. hyopneumoniae* CVCC354 were mainly obtained from China Veterinary Culture Collection Center (CVCC). The *Mycobacterium tuberculosis* H37Ra ATCC25177, H37Rv ATCC27294 and *M. bovis* ATCC19210 were provided by State Key Laboratory of Agricultural Microbiology, Huazhong Agricultural University (Wuhan, China).

IBDV AV150, PRRSV CAU0680, PPV AV31 and CSFV AV1412 were obtained from CVCC. The 50 % tissue culture infective dose (TCID_50_) for the virus was determined by the Reed-Muench assay. The IBDV, PRRSV and PPV were diluted to 1 × 10^-6.25^ (100 TCID_50_), 1 × 10^-4.3^ (100 TCID_50_) and 1 × 10^-4.6^ (100 TCID_50_) respectively with basic medium and stored at −80 °C for future use.

Marc-145 cells, PK-15 cells and DF-1 cells were diluted to 2 × 10^5^ cells/mL with 10 % Dulbecco’s minimum essential medium (DMEM), seeded in 96-well plates, and incubated at 37 °C in a 5 % CO_2_ atmosphere.

### Antimicrobials

CYA, bi-deoxy Cyadox (Cy1), *N*4-deoxy cyadox (Cy2), *N*1-deoxy cyadox (Cy10), QCT, carbonyl reduction bi-deoxy quinocetone (Q2), MEQ, bi-deoxy Mequindox (M1), carbonyl reduction *N*1-deoxy mequindox (M4), carbonyl reduction bi-deoxy mequindox (M5) and carbonyl reduction mequindox (M6) (Table 1) were provided by the National Reference Laboratory of Veterinary Drug Residues, Huazhong Agricultural University (Wuhan, Hubei, China). Amphotericin B, tetracycline, doxycycline, ketoconazole, enrofloxacin, danofloxacin, rifampicin, tilmicosin and kitasamycin were purchased from Dr. Ehrenstorfer (Augsburg, Germany). Kanamycin, pyrazinamide, lincomycin, ethambutol, ribavirin and isoniazid were purchased from TRC (Toront, Canada). Amikacin, clindamycin, and tylosin were purchased from Sigma (St Louis, MO, USA). Stock solutions of the above compounds were prepared at a final concentration of 1280 μg/mL.

**Table 1:**
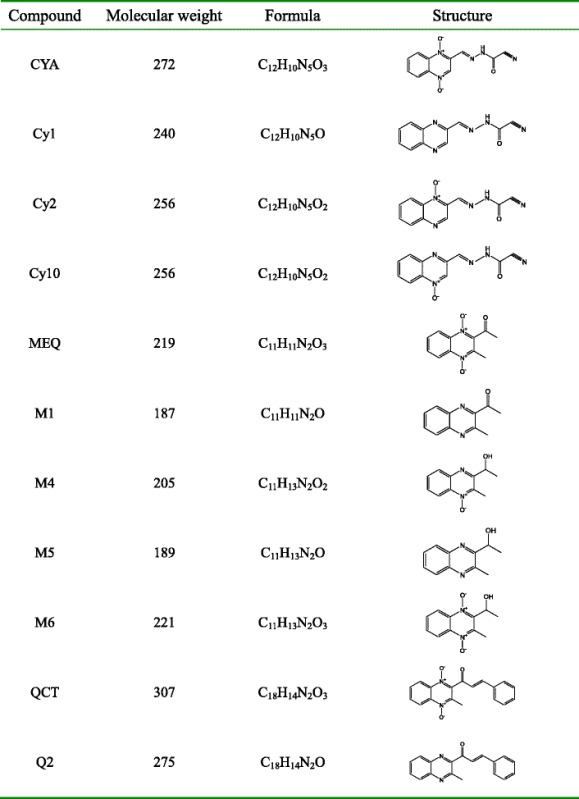
Chemical information of QdNOs and their metabolites

### Reagents

Porcine *mycoplasmas* medium, Middlebrook 7H9 broth base medium, *Mycoplasma gallisepticum* medium and Yeast Peptone Dextrose (YPD) medium were purchased from Qingdao Hope Bio-Technology Co., Ltd (Qingdao, Shandong, China). 1640 medium and horse serum were bought from Gibco (GrandIsland, NY, USA).

DMEM (Hyclone, Beijing, China) supplemented with 10 % or 2 % heat-inactivated fetal calf serum (FCS; Hyclone, USA), 100 IU/ml penicillin G and 100 μg/mL streptomycin was used for cell growth or maintenance medium. A 0.25 % trypsin (Hyclone, Beijing, China) was prepared in pH 7.2 phosphate buffer saline (PBS). A 0.5 % 3-(4,5-dimethyithiazol-2-yl)-2,5-diphenyltetrazolium bromide (Biosharp, Hefei, Anhui, China) was prepared in PBS. These solutions were sterilized through a 0.22 μm Millipore membrane filter. Dimethyl sulfoxide (DMSO) was purchased from Sigma (St Louis, MO, USA).

pMD18-T vector, M-MLV Rtase, Rnasin, Trans1-T1 competent cell, SYBR Premix Ex TaqTM II (Tli RNaseH Plus) were purchased from TaKaRa (Dalian, Liaoning, China). Plasmid Minipreparation Kit and Axyprep DNA Gel Extraction Kit were the products of TIANGEN biotech Co., Ltd (Beijing, China). Trizol Regent was purchased from Ambion (Shanghai, China). All other chemicals and reagents commercially available were of the highest analytical grade.

### Microdilution alamar blue assay (MABA) against *M. tuberculosis* complex

The activities of QdNOs and their metabolites as well as the positive control drugs isoniazide and rifampicin against *M. tuberculosis* complex strains were tested using MABA [18]. Briefly, each of the above *Mycobacterium* strains was cultured at 37 °C in Middlebrook 7H9 broth supplemented with 0.2 % glycerol and 10 % Oleic Acid-Albumin-Dextrose-Catalase (Sigma, St Louis, MO, USA) until logarithmic growth was reached. About 6 × 10^6^ CFU/mL inoculum of *Mycobacterium* strain was then added to the two fold serially diluted drug samples. The final concentration of DMSO in all assays was 2.5 % or less and this dilution also served as solvent control. The samples were assayed in triplicate. All tests were carried out in sterile flat bottom 96-well microplates. Each microplate was incubated for 5 days at 37 °C in a sealed plastic CO_2_-permeable bag. After 7 days of incubation, 32 μL of a mixture of freshly prepared Alamar Blue solution and 20 % sterile Tween-80 at 1:1 (v/v) were added to the growth-control well. The microplates were incubated at 37 °C for 24 h. If a color shift from blue to pink was observed in the growth-control sample, 32 μL of Alamar Blue solution was added to each of the remaining wells, and the microplate was further incubated for 24 h. A well-defined pink color was interpreted as positive bacterial growth, whereas a blue color indicated an absence of growth. The MIC corresponded to the concentration of the greatest dilution of drug sample in which the color shift from blue to pink was not observed.

### Antifungal assay

The antifungal activity of the QdNOs and their metabolites as well as the positive control drugs amphotericin B and ketoconazole were determined according to Rodriguez-Tudela et al. [19]. Briefly, the compounds were tested by macrobroth 2-fold serial dilution technique. *Aspergillus fumigatus*, *Candida albicans*, *Cryptococcus neoformans*, *Candida tropicalis*, *Candida parapsilosis*, *Trichophyton rubrum*, *Epidermophyton floccosum* and *Microsporum canis* seeded broth (10^5^ CFU/mL) were prepared in RPMI 1640 medium, and added into the serially diluted drug solution. The tubes were incubated at 28 °C and the MIC (μg/mL) was recorded after 72 ~ 96 h (mycelial fungi) post-incubation. Broth control (without fungi), growth controls (with fungi and without drug), solvent (DMSO) control and drug control of both test drugs and standard drugs were set under identical conditions. The minimum drug concentration in the tubes in which no apparent growth of the organism was observed represented the MIC of the compound.

### Antimycoplasma assay

The MIC determination of QdNOs and their metabolites and the positive control drugs, tylosin and enrofloxacin, against mycoplasma was performed according to Hannan [20]. Briefly, 96-well microtiter Sensititre plates containing stabilized and freeze-dried antimicrobials were used. Three wells on each plate were set as antimicrobial free growth control. Freshly thawed mycoplasma isolates with known titers were diluted in liquid medium until the number of organisms reached 10^4^ color changing units/mL. 50 μL of the diluted culture was transferred into each well of the Sensititre plates. The *M. gallisepticum* PG31 was used as the control strain and tested three times in order to estimate the reproducibility of the procedure. The plates were sealed using an adhesive foil and incubated at 36 °C for 14 days. The growth of *M. hyopneumoniae* was observed daily when the color of the medium changed from red to yellow (phenol red indicator), and the initial and final MICs were recorded. The initial MIC was defined as the lowest drug concentration at which no change in color when the growth control turned yellow, and the final MIC was defined as the lowest drug concentration to show no color change at 14 days after inoculation.

### Combination susceptibility assay

The fractional inhibitory concentration (FIC) index is most frequently used to describe drug interactions. The combined effects of CYA, MEQ and QCT with antimycoplasma drugs (tetracycline, doxycycline, lincomycin, clindamycin, danofloxacin, enrofloxacin, tylosin and kitasamycin) against *M. gallisepticum* were studied using the checkerboard method [21]. Meanwhile, the combined effects of CYA, MEQ and QCT with antituberculosis drugs (rifampicin, isoniazid, streptomycin, kanamycin, ethambutol, and amikacin) against *M. tuberculosis* H37Rv and *M. bovis* were studied in the same way. FICs were calculated according to the equation: FIC = FIC_A_+ FIC_B_ = A/MIC_A_+ B/MIC_B_, where A and B are the MICs of drug A and drug B in the combination, MIC_A_ and MIC_B_ are the MICs of drug A and drug B alone. Experiments were performed in duplicate. The FIC indices were interpreted as follows: ≤0.5, synergy; 0.5 to 1, additive; 1 to 2, indifferent; >2, antagonism [22].

### Cytotoxicity assay

The cytotoxicity of the QdNO compounds and their metabolites was measured by MTT assay [23]. Each compound or the control drug ribavirin was 2-fold serially diluted with DMEM containing 2 % FCS, respectively. Marc-145 cells, DF-1 cells, and PK-15 cells were seeded into 96-well plates at a density of 2 × 10^4^ cells/well, and incubated for 24 ~ 36 h. When the cells were at least 90 % confluent, the medium was removed and the diluted compounds or ribavirin were added to the wells and incubated for 72 h. Then, the medium was discarded and 20 μL of MTT solution was added to each well. The plates were then further incubated at 37 °C for 4 h. Subsequently, the supernatant was removed and 150 μL of DMSO was added to each well in order to dissolve the formazan crystals. After gently shaking the plates for 10 min, the absorbance was read on an ELISA microplate reader with a 490 nm wavelength and a 630 nm reference wavelength.

For each compound, the percentage of cell viability was calculated as [(A-B)/A × 100], where A and B correspond to the absorbance of control and treated cells, respectively. The 50 % cytotoxic concentration (CC_50_) value was defined as the concentration of each compound that reduced the absorbance of treated cells by 50 % when compared with the non-treated cell control. The maximum non-cytotoxic concentration (MNTC) was calculated as the maximum drug concentration to retain 90 % cell viability [24].

### Antiviral assay

The anti-PRRSV, anti-PPV and anti-IBDV activities of the QdNO compounds and their metabolites were evaluated as previously described by Li et al. [25] with minor modifications. Briefly, a confluent monolayer of cells was prepared as described above. After removal of the culture medium, the MNTC of each compound and a constant amount of 100 TCID_50_ viruses were added. Cell control, virus negative control, and ribavirin positive control were set up simultaneously. The plates were then incubated at 37 °C. When CPE in the virus negative control reached 80 % ~ 90 % compared with cell control, the cell viability was determined by the MTT method. The inhibition ratio was calculated based on the formula [26]: Inhibition ratio = [(OD_T_)_virus_-(OD_C_)_virus_]/[(OD_C_)_mock_-(OD_C_)_virus_], where (OD_T_) _virus_ represents the optical density (OD) of cells infected with virus and treated with the compounds, (OD_C_)_virus_ corresponds to the OD of the untreated virus-infected cells, and (OD_C_)_mock_ is the OD of untreated mock-infected cells. The compounds with the inhibition ratio exceeding 50 % were selected and made by a 2-fold serial dilution with MM, and the procedures were repeated as described above. The 50 % effective concentration (EC_50_) of the compound was defined as 50 % cytoprotection against virus infection. The selectivity index (SI) was calculated as the ratio of CC_50_ to EC_50_. When EC_50_ could not be calculated owing to low inhibition ratio of CPE, the results were counted as the maximum inhibition ratio.

For the virucidal assay [27], each compound with the MNTC and 100 TCID_50_ viruses were mixed and interacted at 37 °C for 2 h. 100 μL of virus/compound suspension was then added to a cell plate and incubated at 37 °C in a 5 % CO_2_ humidified atmosphere. The plate was observed under a microscope daily until the CPE of the virus negative control reached 80 % ~ 90 % compared with cell control, and the MTT test was performed as described above.

The infection inhibition assay was done dynamically according to previous methods [28] with some modifications. The cells in 96-well plates were pre-incubated with 100 TCID_50_ viruses for 2 h. Subsequently, the medium was removed and the cells were washed twice with PBS, and then fresh medium containing MNTC of each compound was added. The plates were further incubated at 37 °C in 5 % CO_2_ atmosphere. The CPE was recorded at a time interval of 12 h under the microscope. When the CPE of the virus negative control reached 80 % ~ 90 % compared with cells control, the anti-virus activity of all phases was assessed by MTT test and the viral inhibition ratio was calculated.

The adsorption inhibition assay was done as followings [29]. The confluent monolayers of cells grown in 96-well plates were incubated with the compounds at 37 °C in a 5 % CO_2_ atmosphere for 4 h. Subsequently, the medium was removed and 100 TCID_50_ viruses were added to each well and incubated for 1 h. The cell monolayer was gently washed with PBS and then fresh medium was added to the plates. The plates were incubated at 37 °C in a humidified atmosphere of 5 % CO_2_ until 80 ~ 90 % CPE was observed in virus negative control compared with cells control. The MTT test and viral inhibition ratio were then determined as above.

### Anti-CSFV assay

CSFV replication is restricted to the cell cytoplasm and does not result in cytopathic effect [30], therefore it was not possible to observe directly the foci of viral growth. Due to this fact, a real-time quantitative PCR (RT-qPCR) using SYBR Green I was developed to determine the copies of virus suspension.

The RNA was extracted from cell culture supernatants of CSFV-infected PK-15 cells using the Trizol method according to the manufacturer’s instructions (Ambion Shanghai, China). The synthesis of cDNA was performed by random priming and using M-MLV reverse transcriptase, as described previously by De Arce et al. [31]. PCR primers (HCLV-FP: 5′-GCAGAAGCCCACCTCGAGAT-3′; HCLV-RP: 5′-TACACCGGTTCCTCCACTCC-3′) synthesized by TIANYI HUIYUAN (Wuhan, Hubei, China) were used to amplify a 244-bp fragment of the conserved 5′-UTR of the genome of hog cholera virus strain HCLV (sequence is available in GenBank no. AF09150). The 50 μL reaction mixture contained 31.0 μL sterilized water, 5.0 μL of 10× buffer, 5.0 μL of dNTP Mixture, 3 μL of MgCl_2_ (25 mmol/L), 1 μL of each primer (HCLV-FP and HCLV-RP), 1 μL of reverse transcription product, and 3 μL of Ex Taq™ DNA Polymerase (Ex taq). The thermal conditions were set as follows: one cycle at 94 °C for 3 min; followed by 35 cycles at 94 °C for 30 s, 59 °C for 30 s, and 72 °C for 30 s; with a final extension at 72 °C for 7 min.

The PCR product was inserted into the vector pMD18-T to construct the recombinant plasmid p-18 T-HCLV which was transformed into in *E. coli* DH5α host bacteria. After increased in the host bacteria, the recombinant plasmid was purified using Plasmid Minipreparation Kit (TIANGEN), and kept at −20 °C for later use.

The real-time PCR amplifications of the target gene fragments used 25 μL reaction mixtures containing 12.5 μL of SYBR premix, 1 μL of cDNA, 0.5 μL of each primer, and 10.5 μL sterile water. The reactions were carried out in BIO-RAD iQ5 Real Time PCR (Hercules, CA, USA). The conditions were as follows: one cycle at 94 °C for 3 min followed by 40 cycles at 94 °C for 30 s, 59 °C for 30 s, and 72 °C for 30 s. Analytical sensitivity was evaluated by testing standard plasmid p-18T-HCLV from sequential ten fold dilutions in DEPC treated water (3.74 ~ 3.74 × 10^8^ copies/μL).

To determine the reproducibility of the real-time PCR, the standard plasmid was diluted to 3.74 × 10^4^, 3.74 × 10^5^ and 3.74 × 10^6^ copies/μL respectively in DEPC treated water. To evaluate intra-assay variability, each dilution was analyzed in triplicate. To measure inter-assay variability, each dilution was analyzed in three different runs performed by two different operators on different days. Coefficients of variation for cycle threshold (Ct) values within each block and among blocks (using the mean values from each block) were determined.

The anti-CSFV assay was done dynamically following previous methods. The MNTC of each compound and CSFV AV1412 were used in the assay. The PK-15 cells in 96-well plates were pre-incubated with CSFV for 2 h. Subsequently, the medium was removed and the cells were washed twice with PBS, and then fresh medium containing the compounds was added. The plates were further incubated at 37 °C in 5 % CO_2_ atmosphere. The cell culture supernatants and CSFV-infected PK-15 cells were collected at 12 h, 24 h, 36 h, 48 h, 72 h, 84 h, 96 h and 108 h respectively. Cells control and virus negative control were set up simultaneously. After RNA isolation and cDNA synthesis, the samples were subjected to the RT-qPCR to detect the copy number. By comparing the growth curve, whether QdNOs have anti-CSFV activity could be judged.

### Statistical analysis

The statistical analysis was performed using the SPSS 19.0 software. Each experiment was repeated three or more times. Data were represented as the means for replicate samples of independent experiments and expressed as the mean ± SD. A student’s t-test and one-way ANOVA were used. A value of *P <* 0.05 was considered statistically significant.

## Results

### Antifungal activity

The results of the in vitro evaluation of antifungal activity of QdNOs are shown in Table 2. The MICs of the quality control drug, amphotericin B and ketoconazole, against the *C. parapsilosis* and *C. albicans* all fell within the same range compared with the results of the previous study [19]. The MIC of CYA against *Cryptococcus neoformans* was determined to be 16 μg/mL and QCT was the most active against *Microsporum canis* with the MIC of 8 μg/mL. The results suggested that QCT could inhibit the growth of superficial fungi and CYA had inhibitory activity against both superficial fungi and deep fungi. The deoxidized metabolites of QdNOs (Table 1) were ineffective against fungi, indicating that the presence of the two *N*-oxide groups in the quinoxaline ring is necessary to the antifungal activity.

**Table 2:** MICs of QdNOs against fungi

					(Unit: μg/mL)	
Fungi		CYA	QCT	MEQ	AMB	KCZ
Superficial fungi	*M. canis* CBS113480	16	8	>64	0.5	0.25
	*M. canis* CMCC(F)M3D	32	16	>64	1	0.5
	*T. rubrum* ATCC4438	32	32	>64	1	0.5
	*T. rubrum* CMCC(F)T1I	32	32	>64	1	0.5
	*E. floccosum* CBS566094	32	32	64	0.5	0.25
	*E. floccosum* CMCC(F)E3D	32	32	>64	0.5	0.25
Deep fungi	*C. albicans* 90028	64	>64	64	1	0.25
	*C. albicans* 2.4122	32	>64	32	2	0.5
	*C. tropicalis* 7349	32	>64	>64	2	0.5
	*C. tropicalis* 2.2735	32	>64	64	2	0.5
	*C. parapsilosis* 22019	32	64	64	1	0.125
	*C. neoformans* 2.3201	16	64	32	0.5	0.25
	*A. fumigatus* 3.5352	32	64	>64	0.5	0.5
	*A. fumigatus* 3.5301	64	64	>64	0.5	0.25

*AMB* amphotericin B, *KCZ* ketoconazole

### Atimycoplasma activity

The antimycoplasma susceptibility test results of 3 QdNOs and 8 antimycoplasma drugs are shown in Table 3. The MICs of the control drugs, tylosin and enrofloxacin, against *M. gallisepticum* and *M. hyopneumoniae* were the same or within 2-fold difference as those in the previous findings [20]. The date obtained indicated the effectiveness of the three QdNO drugs against mycoplasma with MICs between 8 to 32 μg/mL. The MIC of QCT against *M. gallisepticum* was determined to be 8 μg/mL. The metabolites of QdNOs were ineffective against mycoplasma, indicating that the presence of the two *N*-oxide groups ring is necessary to the antimycoplasma activity.

**Table 3:** MICs of QdNOs and other antibacterials against mycoplasma

				(Unit: μg/mL)
	*M. gallisepticum*			*M. hyopneumoniae*
Drugs	BG44T (10^4^ccu/mL)	PG31 (10^8^ ccu/mL)	MG-HS (10^6^ccu/mL)	CVCC354 (10^6^ ccu/mL)
CYA	32	16	16	16
QCT	8	16	16	16
MEQ	16	16	32	32
Tylosin	0.05	0.025	0.025	0.05
Enrofloxacin	0.013	0.025	0.05	0.025
Danofloxacin	0.05	0.05		
Kitasamycin	0.05	0.05		
Tetracycline	0.4	0.8		
Doxycycline	0.1	0.2		
Lincomycin	8	8		
Clindamycin	0.6	3.2		

The MICs of other 8 antimycoplasma drugs showed a good activity against *M. gallisepticum* (Table 3). As shown in Table 4, CYA, MEQ and QCT combined with tetracyclines (tetracycline and doxycycline) had additive effect against *M. gallisepticum*.

**Table 4:** In vitro activity of QdNOs in combination with various antibacterials against *Mycoplasma gallisepticum*

Drug combination	*M. gallisepticum* PG31			*M. gallisepticum* BG44T		
	FIC_A_ + FIC_B_	FIC	Combined effect	FIC_A_ + FIC_B_	FIC	Combined effect
CYA + Tetracycline	0.25 + 0.5	0.75	additive	0.25 + 0.5	0.75	additive
CYA + Doxycycline	0.5 + 0.5	1	additive	0.5 + 0.5	1	additive
CYA + Lincomycin	0.5 + 0.25	0.75	additive	0.25 + 1	1.25	indifferent
CYA + Clindamycin	0.5 + 0.5	1	additive	0.25 + 1	1.25	indifferent
CYA + Danofloxacin	0.5 + 1	1.5	indifferent	0.25 + 1	1.25	indifferent
CYA + Enrofloxacin	0.25 + 1	1.25	indifferent	0.25 + 1	1.25	indifferent
CYA + Tylosin	0.5 + 0.5	1	additive	1 + 0.5	1.5	indifferent
CYA + Kitasamycin	0.25 + 1	1.25	indifferent	0.5 + 0.5	1	additive
QCT + Tetracycline	0.25 + 0.5	0.75	additive	0.25 + 0.5	0.75	additive
QCT + Doxycycline	0.5 + 0.5	1	additive	0.5 + 0.5	1	additive
QCT + Lincomycin	0.5 + 0.5	1	additive	0.25 + 1	1.25	indifferent
QCT + Clindamycin	0.25 + 1	1.25	indifferent	0.25 + 1	1.25	indifferent
QCT + Danofloxacin	0.25 + 1	1.25	indifferent	0.5 + 0.5	1	additive
QCT + Enrofloxacin	0.25 + 1	1.25	indifferent	0.25 + 1	1.25	indifferent
QCT + Tylosin	0.5 + 0.5	1	additive	0.25 + 1	1.25	indifferent
QCT + Kitasamycin	0.25 + 1	1.25	indifferent	0.25 + 1	1.25	indifferent
MEQ + Tetracycline	0.5 + 0.5	1	additive	0.25 + 0.5	0.75	additive
MEQ + Doxycycline	0.5 + 0.5	1	additive	0.5 + 0.5	1	additive
MEQ + Lincomycin	0.25 + 1	1.25	indifferent	0.25 + 1	1.25	indifferent
MEQ + Clindamycin	0.5 + 0.5	1	additive	0.25 + 1	1.25	indifferent
MEQ + Danofloxacin	0.5 + 1	1.5	indifferent	0.25 + 1	1.25	indifferent
MEQ + Enrofloxacin	0.25 + 1	1.25	indifferent	0.25 + 1	1.25	indifferent
MEQ + Tylosin	0.5 + 0.5	1	additive	0.25 + 1	1.25	indifferent
MEQ + Kitasamycin	0.25 + 1	1.25	indifferent	0.5 + 0.5	1	additive

### Antituberculosis activity

The results of antituberculosis activity of QdNOs are shown in Table 5. The MICs of of the quality control drugs (rifampicin and isoniazid) against *M. tuberculosis* H37Rv ATCC27294 were within 2-fold difference range as those in the previous studies [32, 33]. The three QdNO drugs showed good effectiveness against *M. bovis* and *M. tuberculosis* H37Rv with the MICs between 4 to 8 μg/mL. The QdNO metabolites were ineffective against *M. tuberculosis* complex, confirming the findings of the report that the presence of the two *N*-oxide groups in the quinoxaline ring is necessary to the antitubercular activity [34].

**Table 5:** MICs of QdNOs and antituberculosis drugs against *Mycobacterium tuberculosis* complex

		(Unit: μg /mL)	
Drugs	*M. bovis* ATCC 19210	*M. tuberculosis*	
		H37Rv ATCC 27294	H37Ra ATCC 25177
CYA	8	4	64
QCT	4	4	64
MEQ	8	4	32
Rifampicin	0.025	0.05	1.6
Isoniazid	0.025	0.025	16
Streptomycin	0.013	0.025	
Ethambutol	0.025	0.025	
Kanamycin	0.2	0.2	
Pyrazinamide	0.8	0.8	
Amikacin	1.6	3.2	

The results of the MICs of other seven antituberculosis drugs showed a good activity against *M. tuberculosis* complex (Table 5). As can be seen from Table 6, CYA, MEQ and QCT combined with rifampicin had additive effect against *M. tuberculosis* complex with FIC of 0.75.

**Table 6:** In vitro activity of cyadox, quinocetone and mequindox in combination with various antibacterials against *Mycobacterium tuberculosis* complex

Drug combination	*M. bovis* ATCC 19210			*M. tb* H37Rv ATCC 27294		
	FIC_A_ + FIC_B_	FIC	Combined effect	FIC_A_ + FIC_B_	FIC	Combined effect
CYA + Rifampicin	0.5 + 0.25	0.75	additive	0.5 + 0.25	0.75	additive
CYA + Isoniazide	1 + 0.5	1.5	indifferent	1 + 0.25	1.25	indifferent
CYA + Streptomycin	0.5 + 0.25	0.75	additive	0.5 + 0.5	1	additive
CYA + Kanamycin	1 + 0.5	1.25	indifferent	1 + 0.5	1.5	indifferent
CYA + Ethambutol	1 + 0.5	1.5	indifferent	1 + 0.25	1.25	indifferent
CYA + Amikacin	1 + 0.5	1.5	indifferent	1 + 0.25	1.25	indifferent
QCT + Rifampicin	0.5 + 0.5	1	additive	0.5 + 0.25	0.75	additive
QCT + Isoniazide	1 + 0.5	1.5	indifferent	1 + 0.25	1.25	indifferent
QCT + Streptomycin	0.5 + 0.5	1	additive	1 + 0.25	1.25	indifferent
QCT + Kanamycin	1 + 0.5	1.25	indifferent	1 + 0.5	1.5	additive
QCT + Ethambutol	1 + 0.5	1.5	indifferent	1 + 0.25	1.25	indifferent
QCT + Amikacin	0.5 + 0.5	1	additive	1 + 0.5	1.5	indifferent
MEQ + Rifampicin	0.5 + 0.5	1	additive	0.5 + 0.25	0.75	additive
MEQ + Isoniazide	1 + 0.5	1.5	indifferent	1 + 0.25	1.25	indifferent
MEQ + Streptomycin	1 + 0.5	1.55	additive	1 + 0.5	1.5	additive
MEQ + Kanamycin	1 + 0.5	1.5	indifferent	0.5 + 0.5	1	additive
MEQ + Ethambutol	1 + 0.5	1.5	indifferent	1 + 0.5	1.5	indifferent
MEQ + Amikacin	1 + 0.5	1.55	indifferent	1 + 0.5	1.5	indifferent

### Antiviral activity

Cytotoxicity assays are essential for the initial phases of antiviral drug development. The MNTC and CC_50_ values for each tested compound are listed in Table 7. It was observed that CYA, MEQ and QCT exhibited more cytotoxicity than their metabolites to Marc-145 cells, PK-15 cells and DF-1 cells except Q2. The test compounds showed CC_50_ values ranging from 0.81 to 128.62 μg/mL, and the MNTC ranged from 0.06 to 4.0 μg/mL.

**Table 7:** Cytotoxic features of QdNOs and their main metabolites in PK-15, Marc-145 and DF-1 cells

					(Unit: μg/mL, mean ± SD, *n =* 4)	
Drugs	PK-15		Marc-145		DF-1	
	TC_50_	MNTC	TC_50_	MNTC	TC_50_	MNTC
CYA	15.31 ± 1.23	1.2	16.92 ± 1.28	1.56	17.26 ± 1.27	1.0
Cy1	23.40 ± 2.14	2.0	37.83 ± 1.67	3.9	32.97 ± 2.35	2.0
Cy2	51.73 ± 2.96	2.0	96.55 ± 3.12	2.0	35.48 ± 2.87	1.0
Cy10	25.27 ± 1.55	2.0	33.62 ± 1.85	2.0	49.2 ± 1.93	2.0
QCT	2.38 ± 0.27	0.2	8.90 ± 1.05	0.39	9.89 ± 1.08	0.25
Q2	0.81 ± 0.12	0.1	3.76 ± 0.59	0.2	1.86 ± 0.24	0.06
MEQ	17.86 ± 1.28	0.5	41.28 ± 1.28	1.0	40.01 ± 1.27	2.0
M1	72.90 ± 2.66	2.0	70.77 ± 1.57	1.0	54.8 ± 1.93	1.0
M4	40.81 ± 1.27	3.9	128.62 ± 2.67	3.9	37.75 ± 2.68	1.0
M5	40.41 ± 3.21	2.0	89.66 ± 2.14	2.0	38.2 ± 2.09	2.0
M6	78.82 ± 3.57	2.0	89.82 ± 1.57	2.0	41.26 ± 1.27	2.0
Ribavirin	44.80 ± 1.58	2.0	75.49 ± 2.54	8.6	67.42 ± 2.58	4.0

The results obtained from the anti-PRRSV, anti-PPV and anti-IBDV assay demonstrated that QdNOs and their metabolites showed no effectiveness against these viruses in vitro (Additional files 1, 2 and 3). The control drug ribavirin possessed good inhibitory activity in infection inhibition assay, virucidal assay and adsorption inhibition assay.

**Fig. 1:**
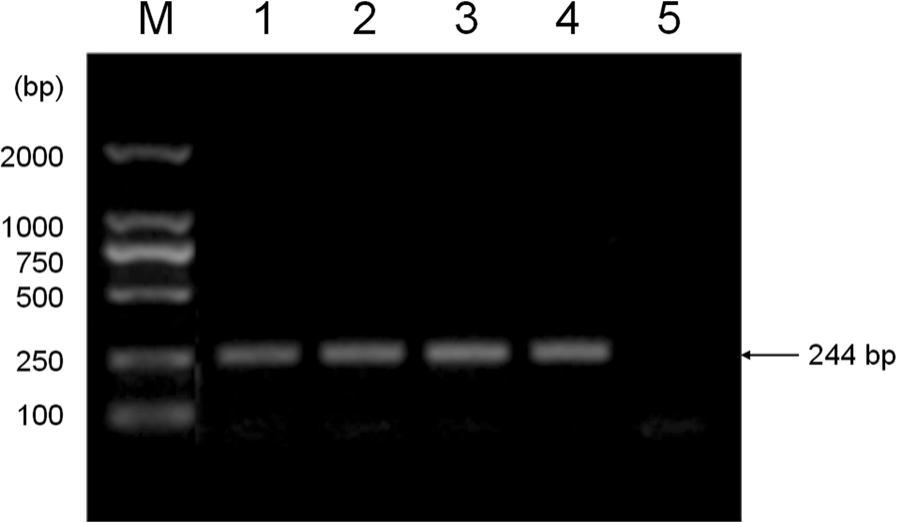
The PCR amplification result of recombinant plasmid p-18T-HCLV. Lane M, DL2000DNA Marker; lane 1 to 4, target fragment; lane 5, blank control

**Fig. 2:**
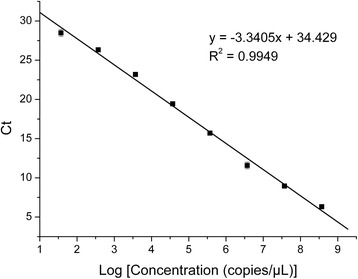
The standard curve for FQ-PCR of standard plasmid. The LogConcentration in the X-axis indicates the denary logarithm of the copy number of standard plasmid per microliter

**Fig. 3:**
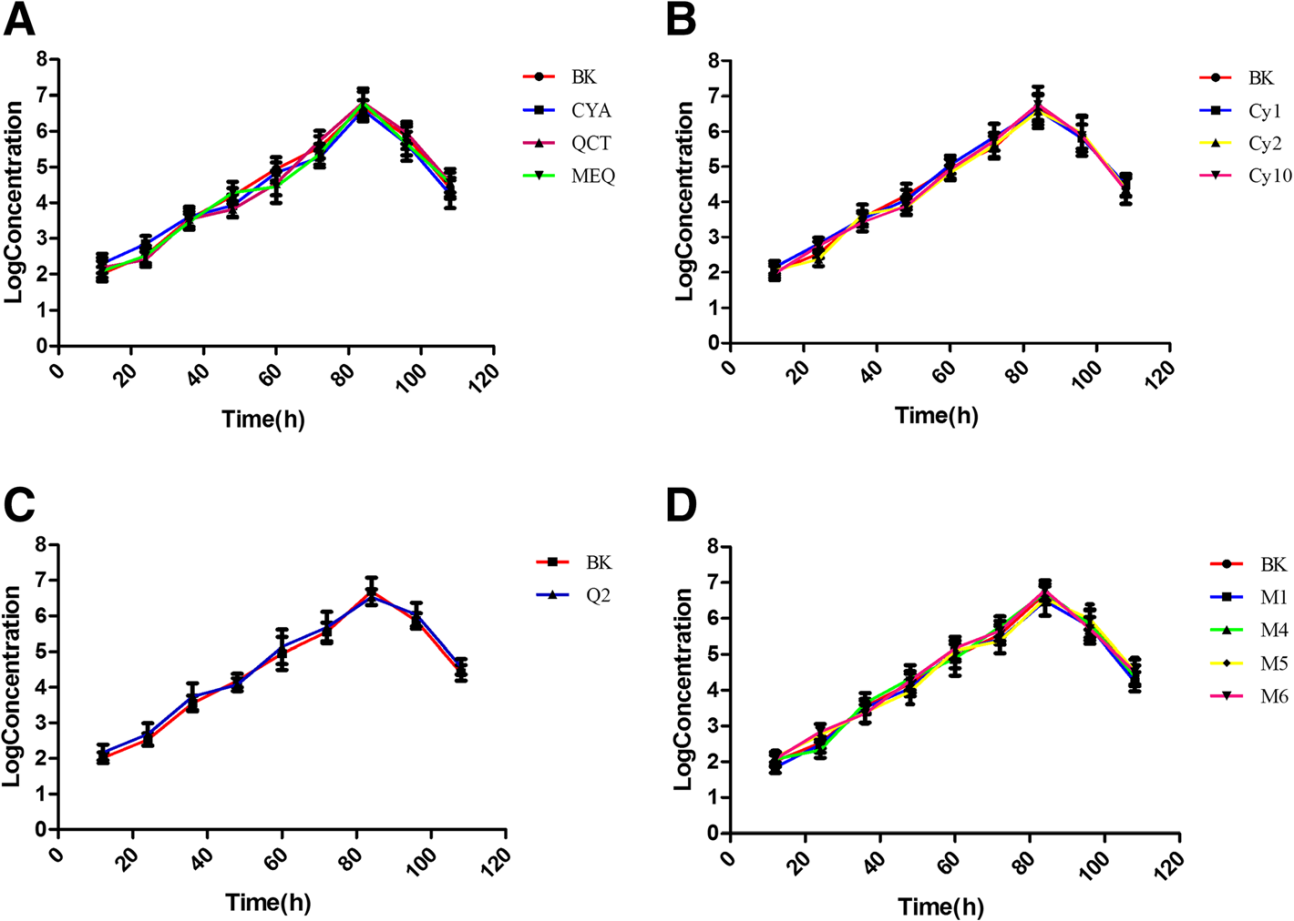
The curve of virus replication after culture with QdNOs and its main metabolites (mean ± SD, *n =* 4). PK-15 cells were incubated with CSFV for 2 h, and then fresh medium containing the compounds (A: CYA, QCT, and MEQ; B: Cy1, Cy2, and Cy 10; C: Q2; D: M1, M4, M5, and M6) was added. The cell culture supernatants and CSFV-infected PK-15 cells were collected at 12 h intervals. After RNA isolation and cDNA synthesis, the samples were subjected to the real-time PCR to detect the copy number. The LogConcentration in the Y-axis indicates the denary logarithm of the copy number of CSFV per microliter. BK, blank control

In the Anti-CSFV assay, the plasmid p-18T-HCLV containing a 244 bp gene fragment of HCLV was used as standard (Fig. 1). The concentration of the plasmid p-18T-HCLV was 1.22 μg/μL before dilution, equivalent to 3.74 × 10^9^ copies/μL. Standard curve were plotted by copy numbers of p-18T-HCLV as the horizontal coordinate and the Ct values as the vertical coordinate based on results of RT-qPCR. The standard curve was linear in the range from 10^9^ to 10^2^ copies/μL, with R^2^ of 0.995 and a reaction efficiency of 99.23 % (Fig. 2). The limit of detection of the RT-qPCR method was 3.74 × 10^1^ copies/μL and the linear range spanned from 3.74 × 10^8^ to 3.74 × 10^1^ copies/μL (Additional file 4). The dissociation curve analysis performed after the completed PCR confirmed only Tm of 86.6 °C for the amplified template (Additional file 5). The amplification plot as well as the melting curve showed nonspecific amplification and non-specific primer dimerization. The amplifications were highly reproducible with coefficients of variation within runs (intra-assay variability) ranging from 0.13 % to 0.80 %, and inter-assay variability ranging from 0.29 % to 0.43 %. By comparing the growth curves we can observe that there are no significant changes of the copies of CSFV between the blank control and the drug-treated group (Fig. 3), indicating that QdNOs have no inhibitory activity against CSFV.

## Discussion

Antonio and coworkers [10] first reported the synthesis and anticandida activities of 36 6(7)substituted-3-methyl-or3-halogenomethyl-2-phenylthio-phenylsulphonyl-chloro-QdNOs. It was shown that the QdNO derivaties without 6(7)-substituted had MIC_50_ of 31.25 μg/mL against 24 clinical-isolated *C. albicans*, consistent with our results (Table 2). Based on the reports and our results, the QdNOs have a general good antifungal activity.

There were few reports on QdNOs against mycoplasma. 23 QdNO derivaties were synthesized and submitted to antimycoplasma assay against *Mycoplasma hominis*, and the results showed several compounds inhibited the growth of the mycoplasma at the concentration of 0.1 mg/mL [16]. Based on our results, the QdNOs may have a better antimycoplasma activity against *M. gallisepticum* an*d M. hyopneumoniae* (Table 3).

The report about the antiviral activity of quinoxalines focused on derivatives of indoloquinoxaline. The antiviral effect of indoloquinoxaline depends on its intercalating into the DNA helix and then disturbing steps that are vital for viral uncoating [14]. The QdNOs were redox-activated, hypoxia-selective DNA cleaving compouds [35]. In our study, neither QdNOs nor their metabolites showed antiviral activity, probably due to the differences in chemical structures of the tested QdNOs and indoloquinoxaline in which the indole groups might be more important for antiviral activity.

Over the past few years, QdNO derivatives have gradually become a research hotspot because they are found to possess good activity against *M. tuberculosis*. What is more, one of the five lead compound series which are currently pursued under the Tuberculosis Antimicrobial Acquisition and Coordinating Facility (TAACF) program is the series of QdNOs derivatives [12]. Over 500 quinoxaline derivatives were tested by the TAACF program, many of these compounds possess good antitubercular activity, and some analogs were even found active against single-drug-resistant strains and non-replicating bacteria [36]. In our study, MEQ showed antitubercular activity against *M. tuberculosis* H37Rv with MIC of 4 μg/mL, in consistence with the previous report (3.13 μg/mL) [11]. The available information so far and our results support the interest devoted to QdNOs as a novel class of antituberculosis agents.

It has been shown that the potency of the quinoxalines make them valid leads for synthesizing new compounds that possess better activity, especially the activity against *M. tuberculosis*. The application of the quantitative structure-activity relationship (QSAR) serves as a rational guide for the design of QdNO derivatives [37]. With the help of the resourceful tool, we can not only synthesize more novel antitubercular candidates, but also modify the old existing quinoxaline drugs.

Synergistic and additive combinations of two or more agents can overcome toxicity and other side effects associated with high doses of single drugs by countering biological compensation, allowing reduced dosage of each compound or accessing context-specific multitarget mechanisms [38, 39]. Combinations of CYA, MEQ and QCT with other antibacterials showed only additive and indifferent interaction with FIC index of 0.75 ~ 1.5. Neither antagonism nor synergism in the QdNO-antibacterial combinations against *M. gallisepticum* and *M. tuberculosis* complex were observed. The results of this in vitro trial provide evidence that CYA, MEQ and QCT, when combined with other antibacterials, could produce a clinically relevant additive effect against these pathogens, without any antagonistic interaction. Therefore, QdNOs may serve as promising compounds for future treatment and prevention of mycoplasmal and tuberculosis infections.

## Conclusion

This study confirmed for the first time that QdNOs have good inhibitory activity against *Mycobacterium tuberculosis* complex and *Mycoplasma*, and they may reduce the threat of drug resistance emerging from those two important pathogens by effective drug combinations. Moreover, this study developed a method for evaluating drugs against CSFV in vitro, providing a new alternative to screen the anti-CSFV drugs. This study gives new insight in further application of QdNOs and offers a way to promote the development of animal husbandry.

## Additional files

Additional file 1: The result of antiviral activity of QdNOs and their metabolites against PRRSV. (DOCX 18 kb) Additional file 2: The result of antiviral activity of QdNOs and their metabolites against PPV. (DOCX 18 kb) Additional file 3: The result of antiviral activity of QdNOs and their metabolites against IBDV. (DOCX 18 kb) Additional file 4: The amplification dynamic curve of RT-qPCR of standard plasmid. (DOCX 342 kb) Additional file 5: The melting curve of RT-qPCR of standard plasmid. (DOCX 168 kb)

## Abbreviations

ATCC: American type culture collection
CC_50_: 50 % cytotoxic concentration
CCU: Colour change unit
CFU: Colony-forming unit
CPE: Cytopathic effect
Ct: Cycle threshold
Cy1: Bi-deoxy cyadox
Cy10: *N*1-deoxy cyadox
Cy2: *N*4-deoxy cyadox
CYA: cyadox
EC_50_: 50 % effective concentration
FIC: Fractional inhibitory concentration index
HCLV: Hog cholera virus strain HCLV
IBDV: Infectious bursal disease virus
M1: Bi-deoxy mequindox
M4: Carbonyl reduction *N*1-deoxy mequindox
M6: Carbonyl reduction mequindox
MABA: Microdilution alamar blue assay
MEQ: Mequindox
MIC: Minimum inhibitory concentration
MNTC: Maximum non-cytotoxic concentration
MTT: Methylathiazol tetrazolium bromide
PPV: Porcine pavrovirus virus
PRRSV: Porcine reproductive and respiratory syndrome virus
Q2: Carbonyl reduction bi-deoxy quinocetone
QCT: Quinocetone
QdNOs: Quinoxaline 1,4-di-*N*-oxides
QSAR: Quantitative structure activity relationship
RT-qPCR: Real-time quantitative PCR
SI: Selectivity index
TAACF: Tuberculosis antimicrobial acquisition and coordinating facility
TCID_50_: 50 % tissue culture infective dose
TI: Therapeutic index

## References

[1] Roe VA (2008). Antibiotic resistance: A guide for effective prescribing in women’s health. J Midwifery Womens Health.

[2] Alanis AJ (2005). Resistance to antibiotics: are we in the post-antibiotic era?. Arch Med Res.

[3] Fernandes R, Amador P, Prudêncio C (2013). β-Lactams: chemical structure, mode of action and mechanisms of resistance. Rev Med Microbiol.

[4] Iland C (1948). Effect of antibacterial analogues of vitamin K on M. tuberculosis. Nature.

[5] Cheng G, Sa W, Cao C, Guo L, Hao H, Liu Z, Wang X, Yuan Z (2016). Quinoxaline 1,4-di-N-Oxides: Biological Activities and Mechanisms of Actions. Frontiers Pharmacol.

[6] Huang Q, Ihsan A, Guo P, Luo X, Cheng G, Hao H, Chen D, Jamil F, Tao Y, Wang X (2016). Evaluation of the safety of primary metabolites of cyadox: Acute and sub-chronic toxicology studies and genotoxicity assessment. Regul Toxicol Pharmacol.

[7] Ihsan A, Wang X, Zhang W, Tu H, Wang Y, Huang L, Iqbal Z, Cheng G, Pan Y, Liu Z (2013). Genotoxicity of quinocetone, cyadox and olaquindox in vitro and in vivo. Food Chem Toxicol.

[8] Hao H, Guo W, Iqbal Z, Cheng G, Wang X, Dai M, Huang L, Wang Y, Peng D, Liu Z (2013). Impact of cyadox on human colonic microflora in chemostat models. Regul Toxicol Pharmacol.

[9] Wang X, Zhou W, Ihsan A, Chen D, Cheng G, Hao H, Liu Z, Wang Y, Yuan Z (2015). Assessment of thirteen-week subchronic oral toxicity of cyadox in Beagle dogs. Regul Toxicol Pharmacol.

[10] Carta A, Paglietti G, Rahbar Nikookar ME, Sanna P, Sechi L, Zanetti S (2002). Novel substituted quinoxaline 1,4-dioxides with in vitro antimycobacterial and anticandida activity. Eur J Med Chem.

[11] Jaso A, Zarranz B, Aldana I, Monge A (2003). Synthesis of new 2-acetyl and 2-benzoyl quinoxaline 1, 4-di-N-oxide derivatives as anti-Mycobacterium tuberculosis agents. Eur J Med Chem.

[12] Vicente E, Villar R, Perez-Silanes S, Aldana I, Goldman RC, Mong A (2011). Quinoxaline 1,4-di-N-oxide and the potential for treating tuberculosis. Infect Disord Drug Targets.

[13] Sainz Y, Montoya ME, Martinez-Crespo FJ, Ortega MA (1999). Lopez dCA, Monge A. New quinoxaline 1, 4-di-N-oxides for treatment of tuberculosis. Arzneimittelforschung.

[14] Harmenberg J, Akesson-Johansson A, Graslund A, Malmfors T, Bergman J, Wahren B, Akerfeldt S, Lundblad L, Cox S (1991). The mechanism of action of the anti-herpes virus compound 2, 3-dimethyl-6 (2-dimethylaminoethyl)-6H-indolo-(2, 3-b) quinoxaline. Antivir Res.

[15] Torres E, Moreno-Viguri E, Galiano S, Devarapally G, Crawford PW, Azqueta A, Arbillaga L, Varela J, Birriel E, Di Maio R (2013). Novel quinoxaline 1, 4-di-N-oxide derivatives as new potential antichagasic agents. Eur J Med Chem.

[16] Carta A, Loriga M, Paglietti G, Mattana A, Fiori PL, Mollicotti P, Sechi L, Zanetti S (2004). Synthesis, anti-mycobacterial, anti-trichomonas and anti-candida in vitro activities of 2-substituted-6, 7-difluoro-3-methylquinoxaline 1, 4-dioxides. Eur J Med Chem.

[17] Hu Y, Xia Q, Shangguan S, Liu X, Hu Y, Sheng R (2012). Synthesis and biological evaluation of 3-aryl-quinoxaline-2-carbonitrile 1, 4-di-N-oxide derivatives as hypoxic selective anti-tumor agents. Molecules.

[18] Jimenez‐Arellanes A, Meckes M, Ramirez R, Torres J, Luna‐Herrera J (2003). Activity against multidrug‐resistant Mycobacterium tuberculosis in Mexican plants used to treat respiratory diseases. Phytother Res.

[19] Rodriguez‐Tudela J, Barchiesi F, Bille J, Chryssanthou E, Cuenca‐Estrella M, Denning D, Donnelly J, Dupont B, Fegeler W, Moore C (2003). Method for the determination of minimum inhibitory concentration (MIC) by broth dilution of fermentative yeasts. Clin Microbiol Infect.

[20] Hannan PC (2000). Guidelines and recommendations for antimicrobial minimum inhibitory concentration (MIC) testing against veterinary mycoplasma species. Vet Res.

[21] Garrod L, Waterworth PM (1962). Methods of testing combined antibiotic bactericidal action and the significance of the results. J Clin Pathol.

[22] Krogstad D, Moellering R (1986). Antimicrobial combinations. Antibiotics Lab Med.

[23] Mosmann T (1983). Rapid colorimetric assay for cellular growth and survival: application to proliferation and cytotoxicity assays. J Immunol Methods.

[24] M-z C, Xie H-g, Yang L-w, Liao Z-h YJ (2010). In vitro anti-influenza virus activities of sulfated polysaccharide fractions from Gracilaria lemaneiformis. Virol Sin.

[25] S-y L, Chen C, H-q Z, Guo H-y, Wang H, Wang L, Zhang X, Hua S-n YJ, P-g X (2005). Identification of natural compounds with antiviral activities against SARS-associated coronavirus. Antivir Res.

[26] Gescher K, Kühn J, Hafezi W, Louis A, Derksen A, Deters A, Lorentzen E, Hensel A (2011). Inhibition of viral adsorption and penetration by an aqueous extract from Rhododendron ferrugineum L. as antiviral principle against herpes simplex virus type-1. Fitoterapia.

[27] Dong C-X, Hayashi K, Mizukoshi Y, Lee J-B, Hayashi T (2011). Structures of acidic polysaccharides from Basella rubra L. and their antiviral effects. Carbohyd Polym.

[28] Alvarez AL, Habtemariam S, Juan‐Badaturuge M, Jackson C, Parra F (2011). In vitro anti HSV‐1 and HSV‐2 activity of Tanacetum vulgare extracts and isolated compounds: An approach to their mechanisms of action. Phytother Res.

[29] Cheng J, Zhao X, Song MQ, Jiang JB, Bai YS, Li HQ (2013). In Vitro Screening for Compounds Derived from Traditional Chinese Medicines with Antiviral Activities Against Porcine Reproductive and Respiratory Syndrome Virus. J Microbiol Biotechn.

[30] Laude H (1987). Hog cholera virus: art and facts. Ann Rech Vet.

[31] De Arce HD, Perez LJ, Frías MT, Rosell R, Tarradas J, Nunez JI, Ganges L (2009). A multiplex RT-PCR assay for the rapid and differential diagnosis of classical swine fever and other pestivirus infections. Vet Microbiol.

[32] Collins L, Franzblau SG (1997). Microplate alamar blue assay versus BACTEC 460 system for high-throughput screening of compounds against Mycobacterium tuberculosis and Mycobacterium avium. Antimicrob Agents Ch.

[33] Ramon Garcia S, Ng C, Anderson H, Chao JD, Zheng X, Pfeifer T, Av-Gay Y, Roberge M, Thompson CJ (2011). Synergistic drug combinations for tuberculosis therapy identified by a novel high-throughput screen. Antimicrob Agents Ch.

[34] Moreno E, Ancizu S, Perez-Silanes S, Torres E, Aldana I, Monge A (2010). Synthesis and antimycobacterial activity of new quinoxaline-2-carboxamide 1, 4-di-N-oxide derivatives. Eur J Med Chem.

[35] Azqueta A, Arbillaga L, Pachón G, Cascante M, Creppy EE, de Cerain AL (2007). A quinoxaline 1, 4-di-N-oxide derivative induces DNA oxidative damage not attenuated by vitamin C and E treatment. Chem-biol Interact.

[36] Gumbo T, Louie A, Deziel MR, Liu W, Parsons LM, Salfinger M, Drusano GL (2007). Concentration-dependent Mycobacterium tuberculosis killing and prevention of resistance by rifampin. Antimicrob Agents Ch.

[37] Vicente E, Duchowicz PR, Castro EA, Monge A (2009). QSAR analysis for quinoxaline-2-carboxylate 1, 4-di-N-oxides as anti-mycobacterial agents. J Mol Graph Model.

[38] Sharom JR, Bellows DS, Tyers M (2004). From large networks to small molecules. Curr Opin Chem Biol.

[39] Keith CT, Borisy AA, Stockwell BR (2005). Multicomponent therapeutics for networked systems. Nat Rev Drug Discov.

