# Supplementary material for: In vitro antimicrobial activities of animal-used quinoxaline 1,4-di-N-oxides against mycobacteria, mycoplasma and fungi
Source: BMC Vet Res. 2016 Sep 6;12(1):186. doi: 10.1186/s12917-016-0812-7 (PMC5011961; doi:10.1186/s12917-016-0812-7)
Supplement: Additional file 2: — The result of antiviral activity of QdNOs and their metabolites against PPV. (DOCX 18 kb) [file 12917_2016_812_MOESM2_ESM.docx]

**Additional file 2 The result of antiviral activity of QdNOs and their metabolites against PPV**

| Drugs | Prevention | |  | Treatment | |  | Inactivation | |
| --- | --- | --- | --- | --- | --- | --- | --- | --- |
|  | IC_50_ (μg/mL) | TI |  | IC_50_ (μg/mL) | TI |  | IC_50_ (μg/mL) | TI |
| CYA | -- | -- | -- | | -- | -- | | -- |
| Cy1 | -- | -- | -- | | -- | -- | | -- |
| Cy2 | -- | -- | -- | | -- | -- | | -- |
| Cy10 | -- | -- | -- | | -- | -- | | -- |
| QCT | -- | -- | -- | | -- | -- | | -- |
| Q2 | -- | -- | -- | | -- | -- | | -- |
| MEQ | -- | -- | -- | | -- | -- | | -- |
| M1 | -- | -- | -- | | -- | -- | | -- |
| M4 | -- | -- | -- | | -- | -- | | -- |
| M5 | -- | -- | -- | | -- | -- | | -- |
| M6 | -- | -- | -- | | -- | -- | | -- |
| Ribavirin | 0.68±0.15* | 65.88 | 0.49±0.07* | | 91.43 | 0.85±0.17* | | 52.71 |

Note: IC_50_ represents as the mean±SD of four independent [experiments](javascript:void(0);); “--” represent not detected owing to the low inhibition ratio; *, *P*<0.05 when compared with the virus control group.

This table provides the QdNOs and their metabolites showed no effectiveness against PPV *in vitro*, while the control drug ribavirin possesses good inhibitory activity in infection inhibition assay, virucidal assay and adsorption inhibition assay.
