# Supplementary material for: In vitro antimicrobial activities of animal-used quinoxaline 1,4-di-N-oxides against mycobacteria, mycoplasma and fungi
Source: BMC Vet Res. 2016 Sep 6;12(1):186. doi: 10.1186/s12917-016-0812-7 (PMC5011961; doi:10.1186/s12917-016-0812-7)
Supplement: Additional file 4: — The amplification dynamic curve of RT-qPCR of standard plasmid. (DOCX 342 kb) [file 12917_2016_812_MOESM4_ESM.docx]

**Additional file 4 The amplification dynamic curve of RT-qPCR of standard plasmid**


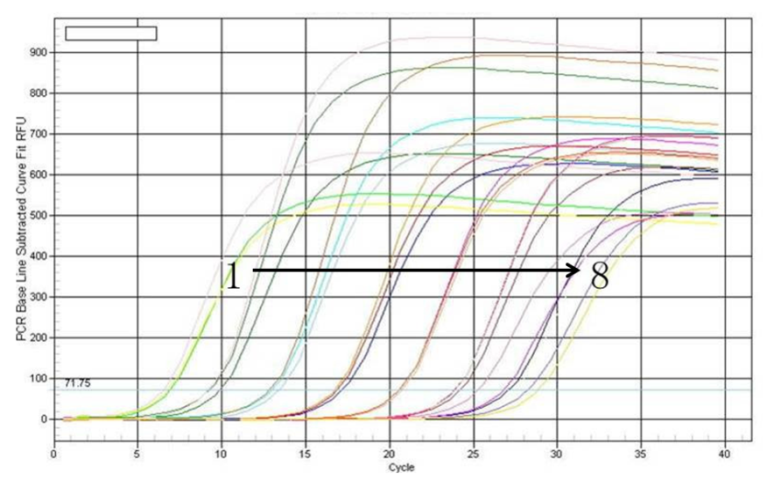


1-8, amplification curves of standard plasmid template with 3.74×10^8^, 3.74×10^7^, 3.74×10^6^, 3.74×10^5^, 3.74×10^4^, 3.74×10^3^, 3.74×10^2^ and 3.74×10^1^ copies/µL，respectively.
