# Supplementary material for: In vitro antimicrobial activities of animal-used quinoxaline 1,4-di-N-oxides against mycobacteria, mycoplasma and fungi
Source: BMC Vet Res. 2016 Sep 6;12(1):186. doi: 10.1186/s12917-016-0812-7 (PMC5011961; doi:10.1186/s12917-016-0812-7)
Supplement: Additional file 5: — The melting curve of RT-qPCR of standard plasmid. (DOCX 168 kb) [file 12917_2016_812_MOESM5_ESM.docx]

**Additional file 5 The melting curve of RT-qPCR of standard plasmid**


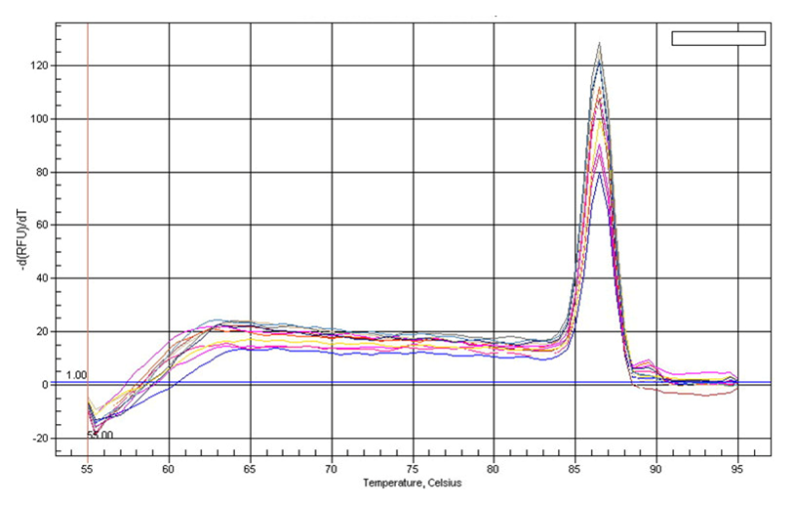


This figure provides only Tm of 86.6°C of the amplified template.
